# Effect of the postoperative pain management model on the psychological status and quality of life of patients in the advanced intensive care unit

**DOI:** 10.1186/s12912-024-02144-z

**Published:** 2024-07-19

**Authors:** Lijuan Wang, Qiang Zhang

**Affiliations:** 1 Department of Rehabilitation Medicine, Pingyi County Hospital of Traditional Chinese Medicine, Linyi, Shandong 273300 China; 2 Department of Critical Care Medicine, Zibo Central Hospital, Zibo, Shandong 255000 China

**Keywords:** Postoperative pain management model, ICU, Psychology, Anxiety, Quality of life

## Abstract

**Objective:**

it was to explore the influence of the postoperative pain management mode on the psychological state, quality of life (QOL), and nursing satisfaction of late patients in the intensive care unit (ICU) and improve the nursing effect of late patients in the ICU.

**Methods:**

seventy patients who were admitted to the postoperative ICU for gastric cancer and received treatment in our hospital from March 2021 to May 2022 were selected. The patients were assigned into a research group and a control (Ctrl) group according to a random number table, with 70 cases in each group. The Ctrl group received routine nursing intervention, while research group received nursing intervention based on routine nursing intervention with postoperative pain management mode and received psychological care. Good communication was established with the patients, and the postoperative pain assessment was well conducted. The general information, state-trait anxiety (STAI) score, World Health Organization’s Quality of Life Instrument (WHO QOL-BREF) score, and care satisfaction were compared.

**Results:**

the general information differed slightly, such as sex, age, and ward type, between groups, with comparability (*P* > 0.05). S-AI scores (13.15 ± 1.53 vs. 16.23 ± 1.24) and T-AI scores (14.73 ± 3.12 vs. 18.73 ± 3.16) in research group were inferior to those in Ctrl group (*P* < 0.05). The scores of patients in research group in the physiological field (78.9 ± 6.1 points vs. 72.3 ± 5.6 points), social relationship field (76.9 ± 4.5 points vs. 71.3 ± 4.8 points), psychological field (78.6 ± 6.2 points vs. 72.4 ± 5.3 points), environmental field (78.6 ± 6.7 points vs. 73.5 ± 6.4 points), and total QOL (79.5 ± 7.4 points vs. 71.6 ± 5.4 points) were higher than those in Ctrl group (*P* < 0.05). The total satisfaction rate with nursing care in research group (82.85%) was dramatically superior to that in Ctrl group (62.85%) (*P* < 0.05).

**Conclusion:**

the adoption of a postoperative pain management model in postoperative nursing interventions for patients in advanced ICUs can alleviate anxiety and depression, improve patients’ QOL and nursing satisfaction, and have clinical promotion value.

## Introduction

The intensive care unit (ICU) is a ward that provides optimal treatment and care for critical patients and can gather critical patients together and use the best human and material support to improve the curative effect of treatment. The basic goal of critical medicine is to ensure the survival of critically ill patients and recover to a certain functional state through etiology and life support treatment [1–4]. Nevertheless, 10–30% of critically ill patients admitted to the ICU die during hospitalization, and a considerable number of survivors leave severe dysfunction and their quality of life (QOL) is also affected [5–8]. At the same time, an increasing number of patients have been treated in the ICU at the end of their lives. According to Western data, the proportion of dead patients admitted to the ICU before their death has increased from 22.4% in 1999 to 29.0% in 2015 [9–12]. These data indicate that ICU medical staff must confront the issue of end-of-life treatment while trying to save the lives of critically ill patients. In addition to routine treatment, appropriate nursing interventions can have a very important impact on the treatment and rehabilitation of ICU patients.

Gastric cancer is a malignant tumor with a high incidence in the clinic. The vast majority of patients with gastric cancer need radical gastrectomy. With continuous changes in diet, many patients will experience a variety of basic diseases, including coronary heart disease, hypertension, and diabetes, which gradually increases the incidence of postoperative complications [13–16]. Therefore, some patients need to be transferred to the ICU for treatment after surgery.

Studies have shown that pain is a complex physiological and psychological state that not only affects the psychological state of patients but also triggers a variety of adverse reactions in the body, seriously affecting the physical and mental health of patients [17–20]. At present, clinical medical staff attach great importance to the adverse effects of postoperative pain for patients in advanced ICUs and are constantly committed to exploring effective means to reduce pain in patients. The pain management mode refers to a pain management group composed of doctors, anesthesiologists, and nurses. By evaluating the vital signs of patients, the group members formulate an appropriate plan and take effective pain intervention measures to improve the pain of patients [21–24].

A total of 70 patients who were transferred to the ICU after surgery for gastric cancer and were treated in our hospital from March 2021 to May 2022 were recruited in this study. They were randomly rolled into a control (Ctrl) group and a research group (35 cases in each group) based on the principle of randomization, aiming to explore the effects of pain management mode on the QOL, psychological state, and nursing satisfaction of patients transferred to the ICU after surgery and to provide a reference for improving the nursing effect of patients in the late ICU.

## Data and methodologies

### Research object

Seventy patients who were transferred to the ICU after gastric cancer surgery and were treated in our hospital from March 2021 to May 2022 were recruited in this study. They were rolled into Ctrl group and research group (35 cases in each group) according to the random principle. There were 19 males and 16 females in Ctrl group whose ages ranged from 45 to 72 years, with an average of 56.3 ± 7.9 years. There were 12 cases of gastric cardia cancer, 8 cases of gastric body cancer, and 15 cases of gastric antrum cancer. The Karnofsky functional status score was 76.8 ± 2.3 points. There were 20 males and 15 females in research group. Their ages ranged from 43 to 73 years old, with an average of 56.7 ± 8.4 years old. There were 13 cases of gastric cardia cancer, 5 cases of gastric body cancer, and 17 cases of gastric antrum cancer. The Karnofsky functional status score was 75.9 ± 2.1 points. No considerable difference was indicated in general information such as sex, age, Karnofsky functional status score, and gastric cancer type between groups (*P* > 0.05), indicating that they were comparable. This study was approved by the Medical Ethics Committee of our hospital. The patients and their families understood the situation of this study and signed the informed consent form.

#### Inclusion criteria

(1) Patients with pathological diagnosis of gastric cancer, which was in line with the diagnostic criteria for gastric cancer; (2) Patients being in normal mental state and able to accurately understand the relevant survey scales; (3) Karnofsy functional status score > 70; (4) Patients who met the surgical indications of radical gastrectomy were transferred to ICU for treatment after surgery.

#### Exclusion criteria

Patients with primary diseases such as heart, liver, and kidney; (2) Patients with nervous system diseases; (3) Karnofsy functional status score ≤ 70; (4) After radical gastrectomy, the patient was transferred to routine ward treatment.

### Intervention measure

Patients in Ctrl group were given routine nursing care, including the detection and recording of various vital signs, on-site nutrition support treatment, health education, psychological intervention, and life care. The patients who suffered from pain were given analgesic drugs according to the doctor’s advice.

Patients in research group were given pain management mode on the basis of routine nursing care.

1. Pain assessment. Based on the patients’ condition and pain status, nursing staff used a professional pain scale to accurately assess the pain severity of the patients.
2. Individualized pain intervention plans were formulated according to the patients’ own situation. According to the above pain assessment results and patients’ tolerance to pain, a personalized pain intervention plan was formulated.
3. Preach about health. Patients were informed of the causes, mechanisms, and influencing factors of postoperative pain and of the advantages of effective analgesia in promoting physical recovery. Medical staff explained the mechanism of action, adverse reactions, and application method of analgesic drugs for patients to improve their understanding of the pain situation and analgesic drugs.
4. Specific implementation of pain intervention measures. (i) Opioid analgesics were given to patients with acute pain after the operation, and analgesic drugs were given according to the doctor’s advice to relieve pain. The adverse reactions of drugs were closely observed during the medication stage. In cases of respiratory depression, malignant vomiting, and urinary retention, medication was stopped immediately. (ii) The patient-controlled analgesia stick was used for effective analgesia, and the patients themselves controlled the administered doses. (iii) Transferring the patient’s attention. According to the patients’ educational level and preferences, music playing and radio listening were used to distract the patients’ attention from the pain. (iv) Cognitive behavioral therapy. Pain health education was conducted for patients. The causes of pain and the effects of pain on the body and psychology were described in detail. The incorrect cognition of patients was eliminated, and the patients’ self-regulation method was informed to minimize the effects of pain. Patients were guided to imagine and self-state, and their feelings of pain were dispersed through positive and optimistic imagination and happy things to strengthen patients’ control over pain. (v) Relaxation therapy. Patients were guided to relax their spirit and whole body muscles by concentrating attention and self-consciousness to relieve anxiety and fear of pain and enhance their tolerance to pain. (vi) Massage. Medical staff massaged the limbs of patients appropriately to help them relax. (vii) Psychological intervention. Medical staff should strengthen communication with patients, understand their psychological state, and tell them successful cases to enhance their confidence in overcoming diseases, urge them to relax, and improve their pain tolerance. For patients with anxiety and pessimism, appropriate psychological counseling should be carried out, emphasizing the role of good mood in reducing pain and accelerating recovery to urge patients to learn self-regulation and actively face the disease.

### Observation indexes

1. General information questionnaire: The investigators designed the questionnaire by themselves to collect and count the general information of patients, including sex, age, Karnofsky functional status score, and type of gastric cancer of the two groups at the time of admission.
2. The visual analog scale (VAS) was employed to assess the pain status of the patients in the two groups on the day of the operation and 1 d, 2 d, and 3 d after the operation. The degree of pain was expressed as a number from 0 to 10. A higher number indicated more severe pain. 0 points meant no pain; 1–3 points meant slight pain, but bearable; 4–6 points meant pain, affecting sleep and tolerable; 7–10 points meant gradually intense pain, unbearable pain, affecting appetite and sleep.
3. World Health Organization’s Quality of Life Instrument (WHO QOL-BREF) scale assessment was performed. The management group used the WHO QOL-BREF to evaluate the QOL of patients in the two groups before and after the intervention. The scale included social function, emotional function, cognitive function, role function, physical function, and other dimensions, all of which were on a 100-point scale. The overall score was the average score of each dimension. The higher the overall score was, the better the QOL of patients.
4. Self-rating anxiety scale (SAS) and self-rating depression scale (SDS) were adopted to evaluate the psychology of patients before and after intervention.
5. The State Trait Anxiety Inventory (STAI) was utilized to assess the negative emotions of patients in the two groups. The STAI is divided into the state anxiety inventory (SAI) and trait anxiety inventory (TAI). The total score was 80 points. The higher the score was, the higher the anxiety was. (The normal values of SAI and TAI were 38.97 and 41.11, respectively).
6. The ICU care satisfaction questionnaire developed by our hospital was adopted to assess the care satisfaction of the two groups, and the calculation of the total satisfaction rate was as follows:

$$\eqalign{& Total{\rm{ }}satiety{\rm{ }}rate{\rm{ }} = {\rm{ }} \cr & \left( {satiety + basic{\rm{ }}satiety} \right){\rm{ }}cases/total{\rm{ }}cases{\rm{ }} \times 100\% \cr}$$

^*^ The questionnaire and scale used in this study has been found on websites, and collected in the supplement file.

### Statistical analysis

Data were analyzed using SPSS 19.0. Measurement data conforming to the normal distribution were denoted as ($\overline{x}$ ± s), and the existence of differences between groups was compared using an independent sample *t* test. The count data were denoted as percentages (%) and tested using χ^2^ test. The difference was statistically significant if *P* < 0.05.

## Results

### General data statistics

In Ctrl group, there were 19 males and 16 females aged 45 to 72 years old, with an average age of 56.3 ± 7.9 years old. The mean course of disease was 3.27 ± 1.25 years, and the body mass index was 24.38 ± 3.27 kg/m^2^. There were 12 cases of gastric cardia cancer, 8 cases of gastric body cancer, and 15 cases of gastric antrum cancer. The Karnofsky functional status score was 76.8 ± 2.3 points. There were 20 males and 15 females in research group, and they were aged from 43 to 73 years old, with an average age of 56.7 ± 8.4 years old. There were 13 cases of gastric cardia cancer, 5 cases of gastric body cancer, and 17 cases of gastric antrum cancer. The Karnofsky functional status score was 75.9 ± 2.1 points. The average course of disease was 3.42 ± 1.26 years, and the body mass index was 25.46 ± 3.28 kg/m^2^. No considerable difference was indicated in general information such as sex, age, disease course, body mass index, Karnofsky functional status score, and gastric cancer type between groups (*P* > 0.05), indicating that they were comparable (Table 1).

**Table 1:** Comparison of general data between groups [$\overline{x}$ ± s, n (%)]

Variables	Ctrl Group	Research Group	*P*-value
**Sex**			
Male	19 (54.3%)	20 (57.1%)	0.728
Female	16 (45.7%)	15 (42.9%)	
**Age (years)**	56.3 ± 7.9	56.7 ± 8.4	0.542
**Course of Disease (years)**			
Male	3.32 ± 1.24	3.49 ± 1.28	0.456
Female	3.23 ± 1.26	3.36 ± 1.24	
**Body Mass Index (kg/m²)**			
Male	24.23 ± 3.19	25.73 ± 3.34	0.112
Female	24.54 ± 3.36	25.22 ± 3.21	
**Types of Gastric Cancer**			
Cardia (n, %)	12 (34.3%)	13 (37.1%)	0.789
Body (n, %)	8 (22.9%)	5 (14.3%)	0.317
Antrum (n, %)	15 (42.9%)	17 (48.6%)	
**Karnofsky Functional Status**	76.8 ± 2.3	75.9 ± 2.1	0.201

### Comparison of VAS pain scores between groups

**Fig. 1:**
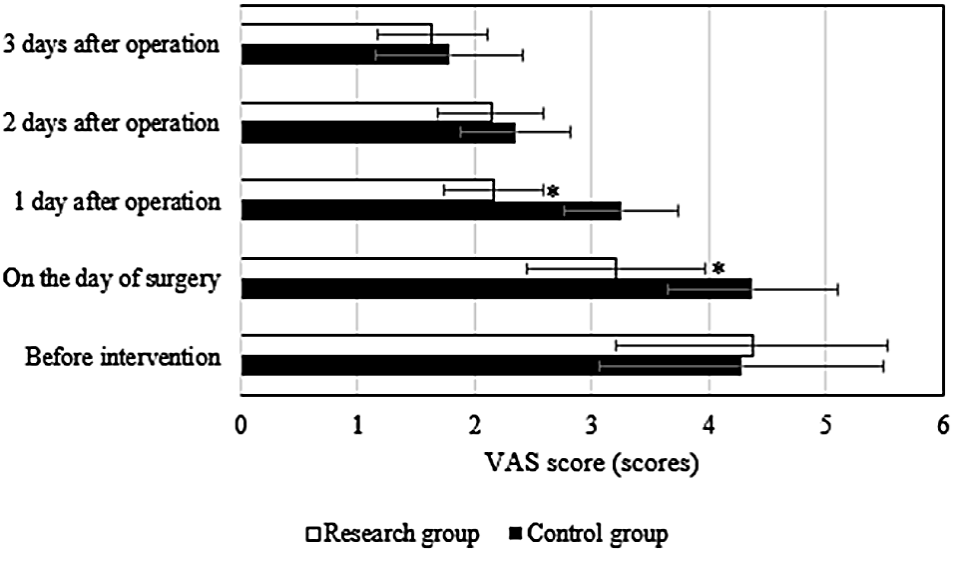
Comparison of VAS pain scores between groups. *Note* * *P* < 0.05 vs. Ctrl group. (* had the same meaning for all the tables and figures below)

The VAS pain scores were compared (Fig. 1). Before the intervention, VAS scores differed slightly between groups (*P* > 0.05). The VAS scores of the two groups on the day of operation in research group were substantially inferior to those of Ctrl group (3.21 ± 0.76 points vs. 4.37 ± 0.73 points), and those of research group on the day after operation were substantially inferior to those of Ctrl group (2.16 ± 0.43 points vs. 3.25 ± 0.48 points), both of which had considerable differences (*P* < 0.05). No considerable difference was indicated in VAS scores on the 2nd and 3rd days after the operation between groups (*P* < 0.05).

### Comparison of QOL before and after intervention between groups

**Fig. 2:**
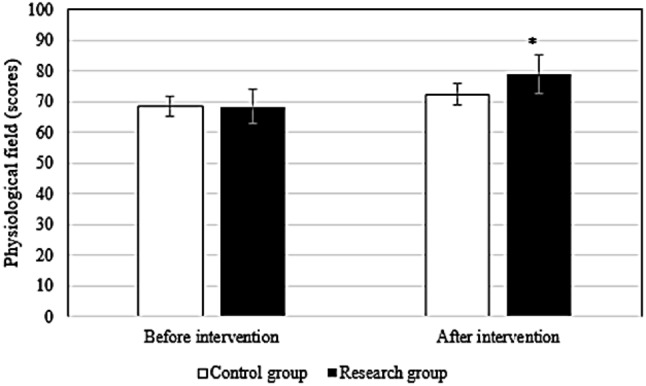
Comparison of physiological fields between groups

**Fig. 3:**
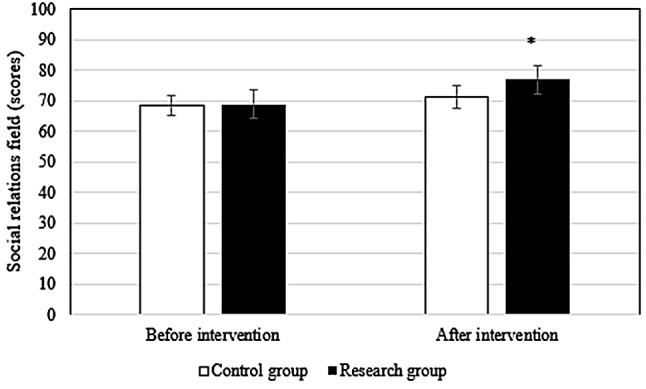
Comparison of social relations between groups

**Fig. 4:**
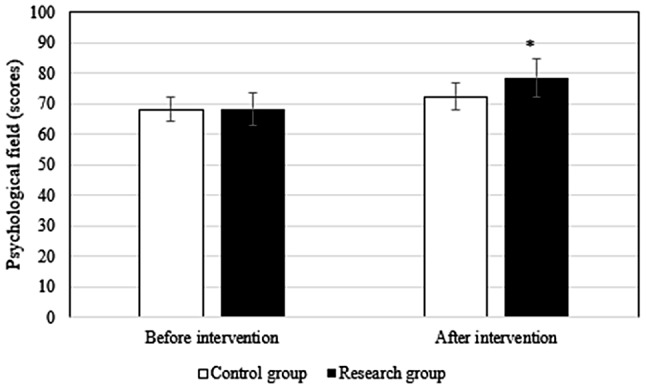
Comparison of psychological field between groups

**Fig. 5:**
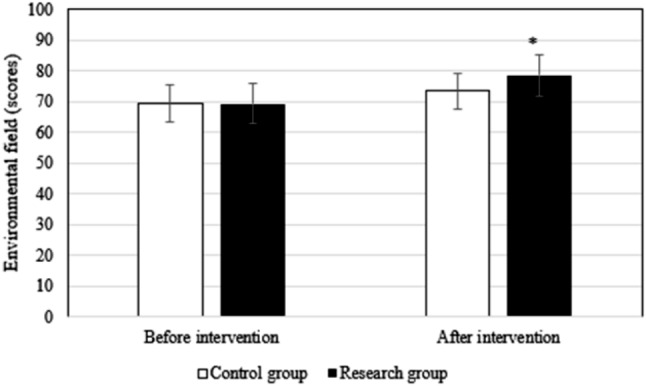
Comparison of the environmental field between groups of patients

**Fig. 6:**
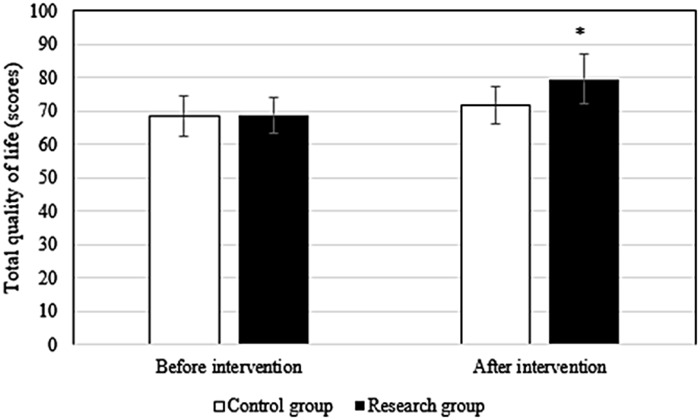
Comparison of total QOL between groups

Before the intervention, the difference in the scores of each field of QOL between groups differed slightly (*P* > 0.05). After intervention, the scores of patients in research group in the physiological field (78.9 ± 6.1 points vs. 72.3 ± 5.6 points), social relationship field (76.9 ± 4.5 points vs. 71.3 ± 4.8 points), psychological field (78.6 ± 6.2 points vs. 72.4 ± 5.3 points), environmental field (78.6 ± 6.7 points vs. 73.5 ± 6.4 points), and total QOL (79.5 ± 7.4 points vs. 71.6 ± 5.4 points) were superior to those in Ctrl group (*P* < 0.05). Figures 2, 3, 4, 5 and 6 show the details.

### Contrast of anxiety and depression scores

Before the intervention, the differences in SAS and SDS scores between groups were not great (*P* > 0.05). After the intervention, the SAS (24.38 ± 5.25 vs. 32.76 ± 5.43) and SDS (24.16 ± 5.27 vs. 31.62 ± 5.36) scores in research group were lower than those in Ctrl group (*P* < 0.05) (Tables 2 and 3).

**Table 2:** Contrast of SAS scores ($\overline{x}$ ± s score)

Item	Ctrl group (*n* = 35)	Research group (*n* = 35)	t	*P*
Preintervention	62.35 ± 7.69	62.37 ± 8.21	8.373	0.625
Postintervention	32.76 ± 5.43	24.38 ± 5.25	3.462	0.004*

**Table 3:** Contrast of SDS scores ($\overline{x}$ ± s score)

Item	Ctrl group (*n* = 35)	Research group (*n* = 35)	t	*P*
Preintervention	62.37 ± 7.58	63.54 ± 8.14	7.235	0.527
Postintervention	31.62 ± 5.36	24.16 ± 5.27	9.153	0.012*

### Comparison of STAI scores before and after intervention between groups

Before the intervention, S-AI and T-AI scores differed slightly between groups within or between groups (*P* > 0.05). After the intervention, the S-AI (13.15 ± 1.53 vs. 16.23 ± 1.24) and T-AI (14.73 ± 3.12 vs. 18.73 ± 3.16) scores in research group were lower versus Ctrl group (*P* < 0.05) (Tables 4 and 5).

**Table 4:** Contrast of S-AI scores ($\overline{x}$ ± s score)

Item	Ctrl group (*n* = 35)	Research group (*n* = 35)	t	*P*
Preintervention	18.25 ± 2.16	17.89 ± 2.14	3.261	0.571
Postintervention	16.23 ± 1.24	13.15 ± 1.53	4.285	0.025*

**Table 5:** Contrast of T-AI scores ($\overline{x}$ ± s score)

Item	Ctrl group (*n* = 35)	Research group (*n* = 35)	t	*P*
Preintervention	20.15 ± 3.27	21.52 ± 3.29	5.285	0.565
Postintervention	18.73 ± 3.16	14.73 ± 3.12	7.943	0.034*

### Contrast of nursing satisfaction

After the intervention, the total satisfaction rate with nursing care in research group (82.85%) was dramatically superior to that in Ctrl group (62.85%) (*P* < 0.05) (Table 6).

**Table 6:** Contrast of nursing satisfaction [n (%)]

Group	Dissatisfied	Basic satisfied	Satisfied	Total satisfaction rate
Ctrl group (*n* = 35)	13 (37.14)	12 (34.28)	10 (28.57)	22 (62.85)
Research group (*n* = 35)	6 (17.14)	13 (37.14)	16 (45.71)	29 (82.85)
χ^2^				5.27
*P*				< 0.05

## Discussion

It has been reported that pain not only restricts the normal activities of the body but also directly affects the sleep and diet of patients, easily causing nutritional disorders of the body, limited mobility, decreased sleep quality, and decreased resistance [25–29]. In addition, pain also affects the psychological reaction of patients, and patients with gastric cancer have various degrees of anxiety, depression, irritability, and other negative emotions, which not only directly affect the QOL of patients but also have a negative impact on the recovery of the disease. Hillier et al. (2022) [30] found that pain control in postoperative gastric cancer patients was affected by a variety of factors, including patients’ emotions, analgesic methods, ward environment, and management level of medical staff. At the same time, patients’ attitude and cognitive level toward pain and analgesic drugs directly affect patients’ ability to control pain and the final analgesic effect. Therefore, the implementation of effective pain management measures for patients in the advanced ICU after gastric cancer surgery has gradually become a hot topic in nursing research.

Postoperative pain assessment is one of the important measures in postoperative pain care and the basis and method of scientific pain care [31–34]. Postoperative pain assessment refers to the measurement and analysis of patients’ postoperative pain and quantification of postoperative pain, which can provide more effective data support for clinical care. Nursing staff used pain measurement tools to measure and record the psychological activities and behavioral performances of patients [35–38]. Meanwhile, changes in respiratory frequency and the degree of psychological anxiety were noticed. The measurement of postoperative pain in clinical practice can not only enable patients to understand their own pain situation but also provide a reference for nursing staff to carry out scientific and reasonable assessments of postoperative pain.

A pain management group composed of doctors, anesthesiologists, and nurses was established in our hospital. The group comprehensively evaluated the vital signs of patients through the evaluation of their resting state and strengthened the management of patients according to their postoperative pain, aiming to reduce their postoperative pain. In this study, pain management was applied to the care of patients in the advanced stage in the postoperative ICU, and a good nursing effect was demonstrated. It was revealed that the VAS scores of patients in research group on the day after the operation and the first day after the operation were lower versus Ctrl group (*P* < 0.05). These results indicated that the implementation of a pain management model could evidently reduce pain in patients, promote the healing of surgical incisions, and help patients recover as soon as possible. The possible reasons were as follows. First, preemptive analgesia before surgery mainly involves analgesic drugs, mainly nonsteroidal anti-inflammatory drugs, which undergo a series of physiological changes to achieve analgesic effects. Second, the pain management mode enables patients to receive comprehensive and timely pain management in the whole process from admission to discharge. At the same time, effective preoperative and postoperative psychological counseling can evidently reduce pain caused by the psychological stress response and evidently improve the postoperative analgesic effect of patients.

The lack of good communication with patients is often one of the reasons for errors in clinical care; therefore, good communication with patients and their techniques is needed in advanced ICU patients. Nursing staff should enable patients to establish a correct understanding of postoperative pain, analgesic drugs, and pain care measures and understand and support pain assessment methods in clinical care. On the premise of patients’ understanding and support, the difficulty of nursing work was reduced [39–41]. In addition, establishing good communication with patients was conducive to the development of auxiliary care. Medical staff should guide patients to carry out relevant pain rehabilitation activities to let patients understand the methods to reduce pain [42–45]. A small number of patients have prejudice toward the use of analgesics, and they should have good communication so that they can understand the use of analgesics and their disadvantages, illustrating the importance of analgesic measures in perioperative care.

In this study, the QOL score of research group was dramatically superior to Ctrl group (*P* < 0.05), suggesting that the pain management model could effectively promote the postoperative recovery of late ICU patients and improve the QOL of patients. Possible reasons were as follows. First, the pain management model implements health promotion for patients, which improves patients’ understanding of the disease, enables patients to identify the impact of pain on prognosis, promotes patients to form a good living habit, fully mobilizes patients’ enthusiasm for self-management, promotes patients’ recovery from surgical incision, and improves patients’ QOL. Second, the health care concept of the pain management group was enhanced so that the members of the health care group paid attention to the complaints of patients and paid attention to pain education and could accurately assess the pain in patients and formulate personalized treatment plans, which improved the effect of postoperative analgesia.

Due to the inevitability of postoperative pain, it is very important to take good psychological intervention measures for patients. After the operation, patients often suffer from depression and anxiety due to postoperative pain, and their confidence in the rehabilitation of their own condition and surgical treatment will also be affected [46, 47]. Therefore, adopting the pain nursing mode of appropriate psychological intervention can provide reasonable support to patients, let patients understand the generality of postoperative pain, and reduce the psychological burden of patients. At the same time, the nursing staff should create a good ward environment and keep the ward quiet and tidy so that patients can more actively receive the guidance of the medical staff and reduce psychological anxiety to a great extent.

Persistent pain produces negative emotions and triggers other clinical symptoms, such as poor appetite and loss of happiness; to a certain extent, it directly affects the QOL of patients [48]. The results revealed that SAS and SDS scores between groups differed slightly before intervention, while the difference in SAS and SDS scores between groups after intervention was great (*P* < 0.05). This result indicated that the pain management mode intervention in the study changed the patients from passive to active psychological cognitive ability so that they could obtain better postoperative care and reduce the harm caused by the mentality of patients.

According to the research by McKenzie et al. (2021) [49], analgesic knowledge education can evidently improve patients’ cognition of pain and their demand for postoperative analgesia, which is conducive to improving their acceptance of postoperative analgesia, promoting their control over pain, and improving their postoperative QOL. The results further confirmed the above views. In addition, it was found that the pain management mode improved the patients’ cognition of pain knowledge and was conducive to their actively cooperating with the medical staff in treatment and care, self-regulating negative emotions, and improving their hopes for disease recovery, thereby further promoting the recovery of patients’ physiological functions and improving their QOL. The results showed that before and after the intervention, the difference in STAI score between research group and Ctrl group was great (*P* < 0.05), indicating that the pain management model could effectively improve the negative emotions of late ICU patients, which was related to the promotion of the pain management model to relieve pain in patients. The nursing satisfaction of patients in research group was dramatically superior to that of Ctrl group (*P* < 0.05), indicating that the pain management mode was conducive to improving the nursing quality of patients in the late ICU and nursing satisfaction.

In summary, the application of a postoperative pain management model in postoperative nursing interventions for patients in advanced ICUs can alleviate anxiety and depression, improve patients’ QOL and nursing satisfaction, and have clinical promotion value [50].

Also, a number of limitations were identified. The sample size of 70 patients from a single centre may limit the generalisability of the findings. The selection bias may have favoured healthier patients without specifying the comorbidities. The short-term follow-up did not assess the long-term impacts. The intervention details were vague, impeding replication efforts. The reliance on subjective self-report may have introduced bias. The reports of outcomes introduced potential bias, as the handling of missing data and dropouts was not clarified. The blinding status was unclear, which risked assessment bias. The control group’s “routine nursing” was undefined, which complicated the isolation of the intervention effect. Finally, the cultural context, specific to China, could have influenced the results. It is recommended that future studies consider multicentre designs, larger samples, longer follow-ups, detailed intervention protocols, objective measures, clearer definitions of control interventions, blinding, and account for cultural diversity in order to strengthen the findings and applicability of the results. [51–53]

## Conclusion

The application of a postoperative pain management model in postoperative nursing interventions for patients in advanced ICUs can alleviate anxiety and depression, improve patients’ QOL and nursing satisfaction, and have clinical promotion value. Nevertheless, the shortcomings of this study lie in that the subjects came from only one hospital, with geographical and cultural bias, and needed to be appropriately adjusted in the late stage to promote comprehensive nursing intervention. Moreover, due to the objective restrictions on funding, venue, and personnel, the subjects in this study were limited, and we hope that the scope of in-depth evaluation can be expanded in future studies.

## Data Availability

The figures and tables used to support the findings of this study are included in the article.
